# Supplementary material for: Physics-informed deep learning approach for modeling crustal deformation
Source: Nat Commun. 2022 Nov 19;13:7092. doi: 10.1038/s41467-022-34922-1 (PMC9675849; doi:10.1038/s41467-022-34922-1)
Supplement: Supplementary file 1 — Supplementary Information [file 41467_2022_34922_MOESM1_ESM.pdf]

Supplementary Material for

Physics-Informed Deep Learning Approach for Modeling Crustal Deformation

Tomohisa Okazaki[1], Takeo Ito[2], Kazuro Hirahara[1], Naonori Ueda[1]

[1]RIKEN Center for Advanced Intelligence Project, Japan
[2]Graduate School of Environmental Studies, Nagoya University, Japan

Supplementary Text 1. To validate the accuracy of PINN solutions for the curved, heterogeneous models in Figure 3, we constructed simple three-dimensional FEM models using the PyLith software 2.2.2[1]. The model space should be sufficiently large to suppress boundary effects and was set as 150, 21, and 100 in the x, y, and z (antiplane) directions, respectively, in the normalized length scale used in the PINN modeling. In mesh generation, we used tetrahedron cells that were denser near the fault and ground surfaces. The smallest and largest cell sizes were approximately 0.03 and 3.0 scales, respectively. As a result, the number of cells and nodes was 790,392 and 138,110, respectively. We set up the pure Dirichlet boundary condition (roller condition) on the positive and negative x-faces and the negative y-face. The roller condition constrained the displacement in a direction normal to each face. To simulate the fault behavior, we prescribed a kinematic slip corresponding to a relative motion across the fault surface.

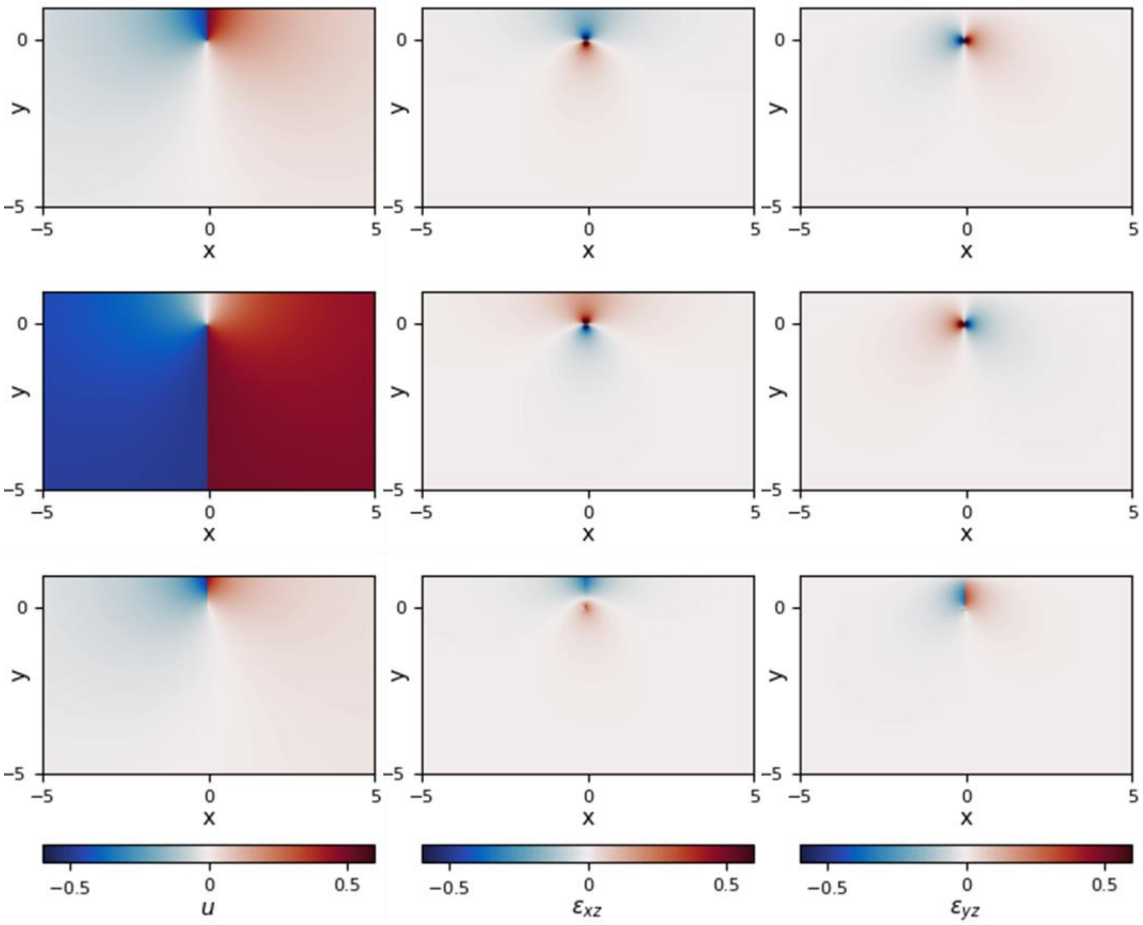

Supplementary Figure 1. Displacement (left), strain components ε_{xz} (center), and ε_{yz} (right) of the homogeneous models shown in Figure 2. The top, middle, and bottom rows show Models 1A, 1B, and 1C, respectively.

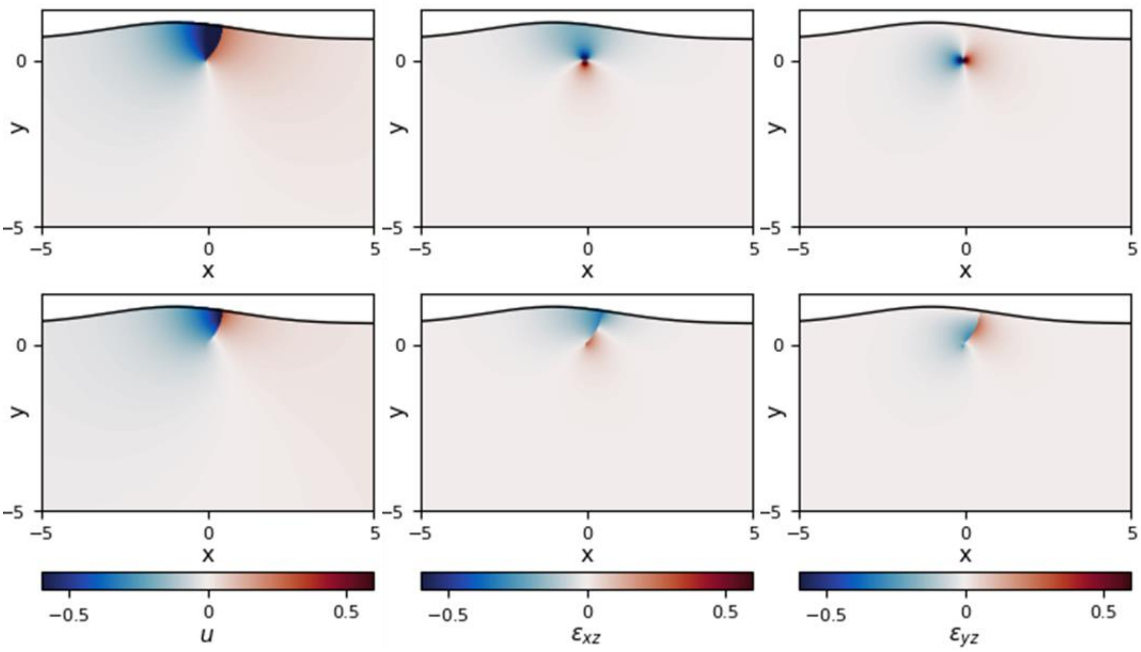

Supplementary Figure 2. Displacement (left), strain components ε_{xz} (center) and ε_{yz} (right) of the heterogeneous models shown in Figure 3. The top and bottom rows show Models 2A and 2B, respectively.

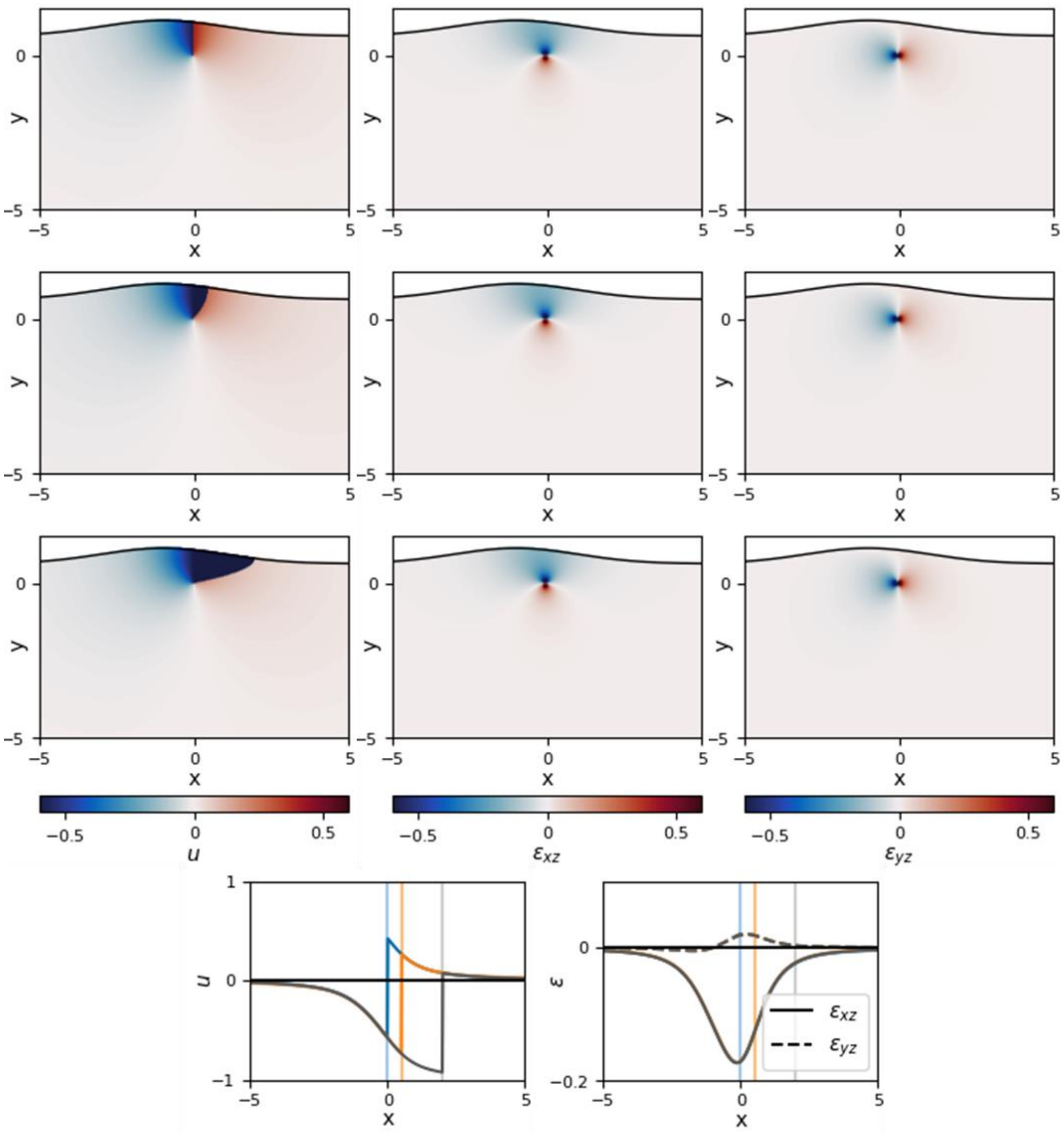

Supplementary Figure 3. Displacement (left), strain components ε_{xz} (center), and ε_{yz} (right) of three fault geometries in a heterogeneous medium. The fault geometries are distinguished by displacement discontinuity in the left panels. The surface topography and mechanical properties are the same as those in Figure 3a. Uniform slips are assumed on the faults. The bottom row shows surface displacement (left) and strain (right). Blue, orange, and gray lines correspond to the results of the first, second, and third rows, respectively. Vertical lines indicate fault locations.

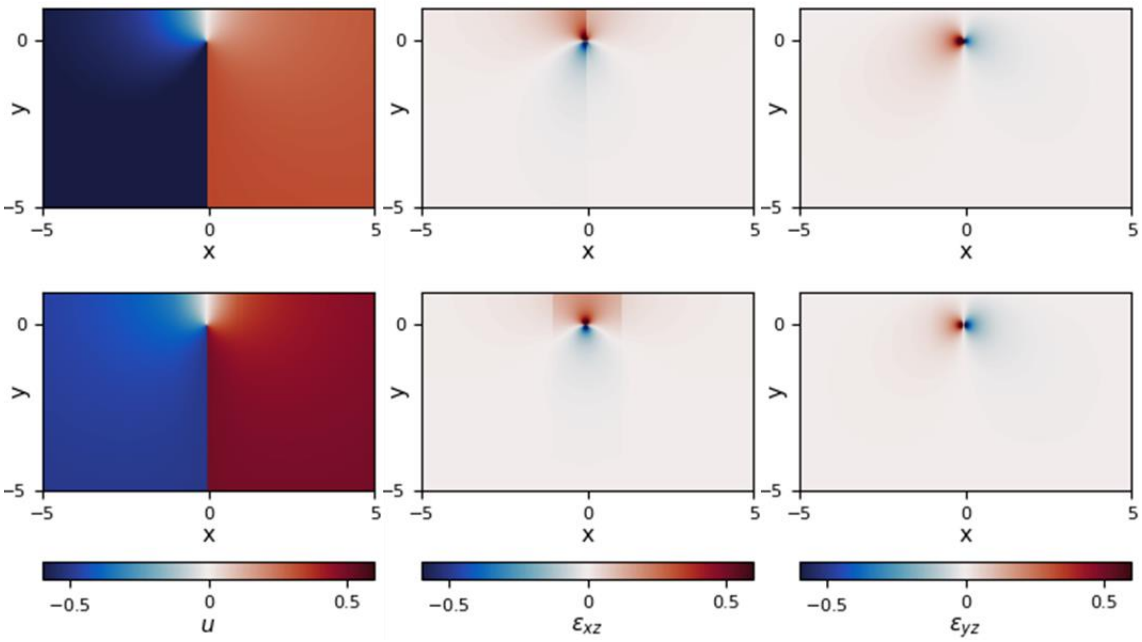

Supplementary Figure 4. Displacement (left), strain components ε_{xz} (center), and ε_{yz} (right) of the discontinuous material models shown in Figure 4. The top and bottom rows show Models 3A and 3B, respectively.

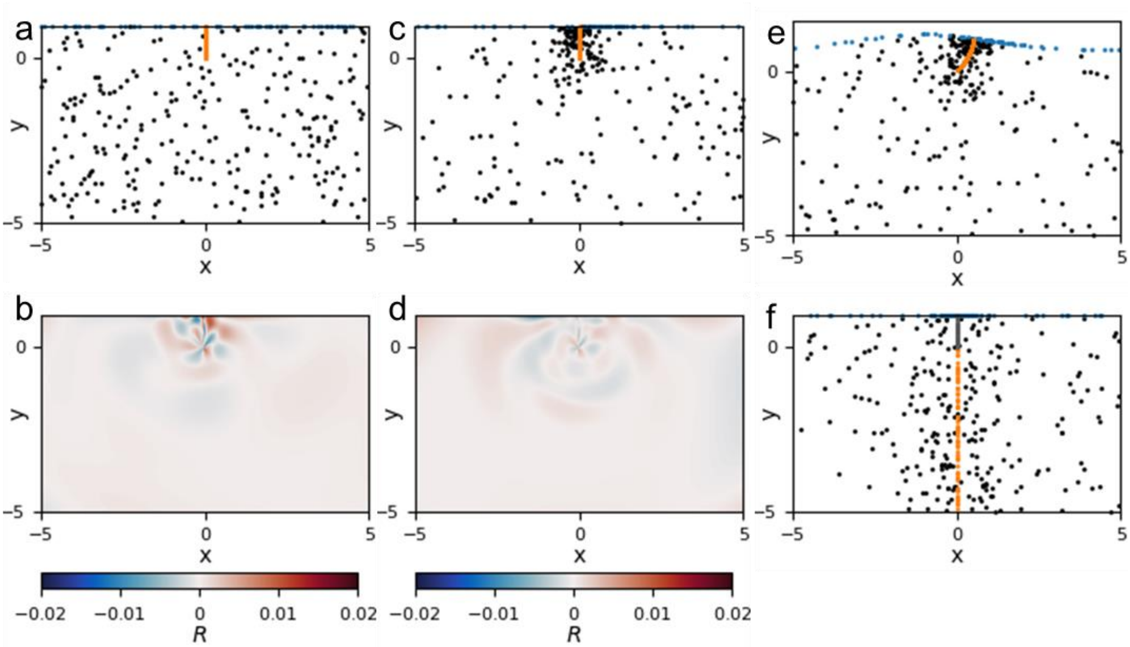

Supplementary Figure 5. Examples of collocation points and distribution of residuals. (a) Uniform sampling in a homogeneous half-space. (b) Distribution of residuals for a neural network trained with the uniform sampling in (a). (c) Concentrated sampling in a homogeneous half-space. (d) Distribution of residuals for a neural network trained with the concentrated sampling in (b). (e) Concentrated sampling of a model in Figure 3. (f) Concentrated sampling of a model in Figure 4. Black, orange, blue, and gray dots represent the collocation points in V and on Σ, S, and B, respectively.

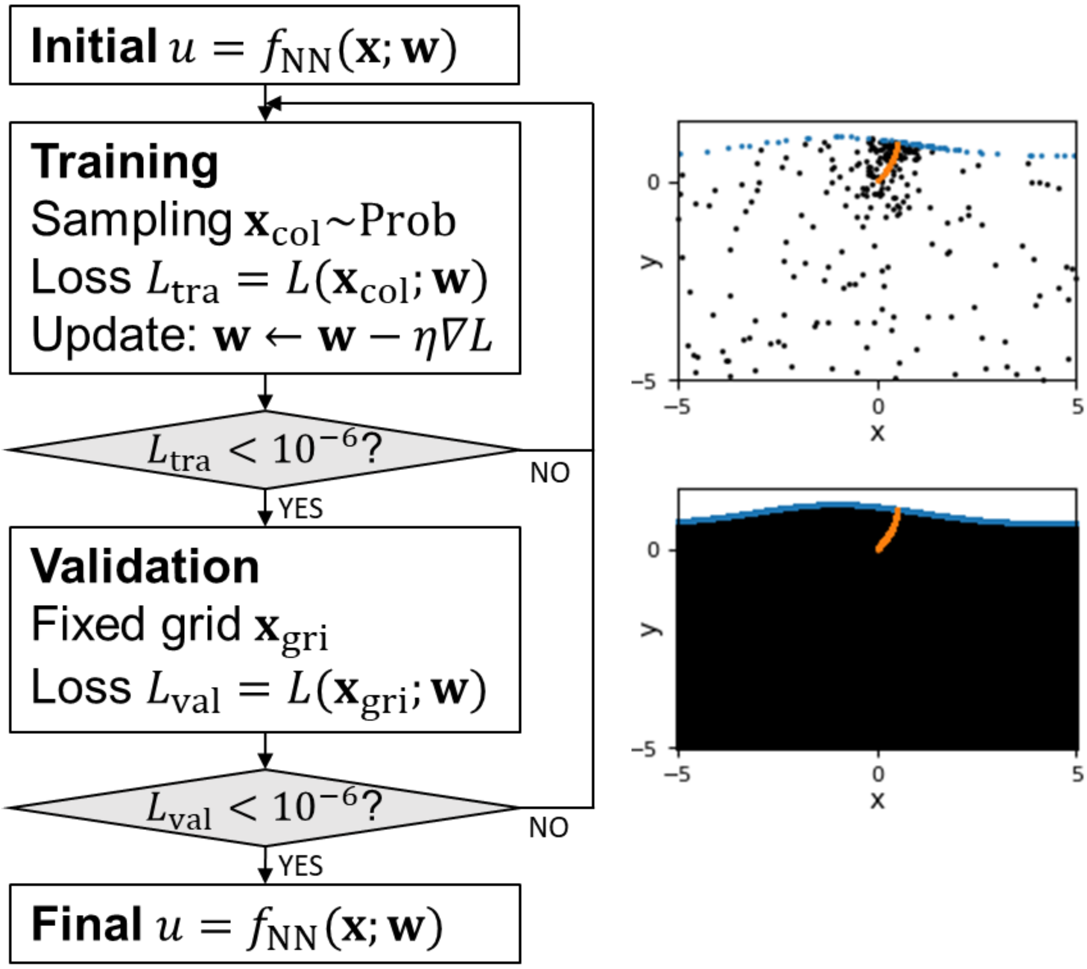

Supplementary Figure 6. Optimization procedure. Left: Flow chart of the training. Training is iterated until both $L_{tra} < 10^{-6}$ and $L_{val} < 10^{-6}$ are satisfied. Right: Distributions of collocation points $\mathbf{x}_{col}$ (top) and fixed grid points $\mathbf{x}_{gri}$ (bottom) for a model in Figure 3. Black, orange, and blue dots represent collocation points in V and on Σ and S, respectively.

Supplementary References

1. Aagaard, B. T., Knepley, M. G., & Williams, C. A., A domain decomposition approach to implementing fault slip in finite-element models of quasi-static and dynamic crustal deformation. Journal of Geophysical Research: Solid Earth, 118, 3059–3079 (2013).
