# Supplementary material for: Physics-informed deep learning approach for modeling crustal deformation
Source: Nat Commun. 2022 Nov 19;13:7092. doi: 10.1038/s41467-022-34922-1 (PMC9675849; doi:10.1038/s41467-022-34922-1)
Supplement: Supplementary file 2 — Description of Additional Supplementary Files [file 41467_2022_34922_MOESM2_ESM.pdf]

Description of Additional Supplementary Files

Supplementary Software 1: Source programs of physics-informed neural networks for antiplane dislocation models. The readme file includes the parameter setting for the models in the article.
